# Conversion of orange peel to L-galactonic acid in a consolidated process using engineered strains of *Aspergillus niger*

**DOI:** 10.1186/s13568-014-0033-z

**Published:** 2014-03-18

**Authors:** Joosu Kuivanen, Hugo Dantas, Dominik Mojzita, Edgar Mallmann, Alessandra Biz, Nadia Krieger, David Mitchell, Peter Richard

**Affiliations:** 1 VTT Technical Research Centre of Finland, Espoo, 02044 VTT, Finland; 2 Department of Biochemistry and Molecular Biology, Federal University of Paraná, Curitiba-PR, 81531-990, Brazil

**Keywords:** Citrus processing waste, Orange peel, Consolidated bioprocessing, L-galactonic acid, D-galacturonic acid, Aspergillus niger

## Abstract

Citrus processing waste is a leftover from the citrus processing industry and is available in large amounts. Typically, this waste is dried to produce animal feed, but sometimes it is just dumped. Its main component is the peel, which consists mostly of pectin, with D-galacturonic acid as the main monomer. *Aspergillus niger* is a filamentous fungus that efficiently produces pectinases for the hydrolysis of pectin and uses the resulting D-galacturonic acid and most of the other components of citrus peel for growth. We used engineered *A. niger* strains that were not able to catabolise D-galacturonic acid, but instead converted it to L-galactonic acid. These strains also produced pectinases for the hydrolysis of pectin and were used for the conversion of pectin in orange peel to L-galactonic acid in a consolidated process. The D-galacturonic acid in the orange peel was converted to L-galactonic acid with a yield close to 90%. Submerged and solid-state fermentation processes were compared.

## Introduction

Utilization of agricultural wastes for the production of fuels and chemicals using microbial fermentations is an attractive concept, due to the low costs of the raw material and beneficial environmental aspects. However, the profitability of utilizing agricultural residues can be reduced by logistical costs, if the feedstock is dispersed over a wide area and by the need for pre-treatments before the fermentation. Consequently, the ideal process would utilize an existing logistic chain and a feedstock that does not need extensive pre-treatment. Citrus processing waste (CPW) is an example of such a feedstock: its generation is centralized within a citrus processing plant and, due to low lignin content (Edwards and Doran-Peterson [2012]), it does not require extensive pre-treatment.

The current annual world production of citrus fruits is greater than 80 million tonnes, of which oranges constitute about 50 million tonnes (USDA [2013]). About 20 million tonnes of these oranges are used by the orange processing industry (USDA [2013]). The production of orange juice generates a large quantity of CPW, with around 44–60% of the mass of processed orange fruit ending up as CPW (Widmer et al. [2010], López et al. [2010]). Thus, approximately 10 million tonnes of wet CPW is generated annually in the world by the orange processing industry alone.

CPW, in turn, contains mainly peels. These peels are rich in pectin and also contain a significant quantity of D-limonene. The pectin content in CPW is around 25% on a dry mass basis, corresponding to about 5% on a wet mass basis (Pourbafrani et al. [2010]). Currently, the markets for pectin and D-limonene consume only a relatively small fraction of the CPW. For example, the world market for pectin, which is used as a gelling agent in the food industry, was estimated as 42 000 tonnes per year in 2009 (Staunstrup [2009]), whereas the CPW generated annually by the orange processing industry could produce approximately 500 000 tonnes of pectin. In other words, over 90% of the CPW produced by the orange processing industry is in excess of the world pectin market. This excess CPW could be used for animal feed. However, this is seldom economic, due to the high energy requirements for the processes of drying and milling that are necessary to transform the CPW into a meal that is appropriate for adding to animal feed (Grohman et al. [2013]). As a result, the CPW is sometimes simply discarded. It is therefore highly desirable to find new ways of converting pectin-rich residues into useful products, following the worldwide trend of using renewable resources to substitute petroleum derivatives.

So far, most attempts to produce higher-value products from CPW have involved extracting compounds such as pectin and D-limonene and fermenting the remaining matter to produce ethanol (Edwards and Doran-Peterson [2012]). However, due to the limited size of the world pectin market and the inability of ethanologenic yeasts to ferment D-galacturonic acid (Van Maris et al. [2006]), the main constituent in pectin, it is possible to utilize only a part of CPW in these processes. Other proposed products from CPW include methane (López et al. [2010]), citric acid (Rivas et al. [2008]), succinic acid (Li et al. [2010a]) and enzymes (López et al. [2010]). In the current work, we open a new route to increase the value of CPW using engineered *Aspergillus niger* strains to convert the pectin to L-galactonic acid in a consolidated process.

L-Galactonic acid and its lactone (L-galactono-1,4-lactone) are currently expensive speciality chemicals and are not widely used. However, these compounds would have the potential to be used more widely if they were available at a lower price. L-Galactonic acid is also a precursor for L-ascorbic acid (vitamin C): L-galactono-1,4-lactone, which is formed from L-galactonic acid upon acidification, can be directly converted to L-ascorbic acid through a fermentative process (Onofri et al. [1997], Roland et al. [1986]) or through a chemical process (Csiba et al. [1993]). The annual production of synthetic L-ascorbic acid is about 100 000 tonnes (Pappenberger and Hohmann [2013]). The 500 000 tonnes of pectin in the annually produced CPW contains around 375 000 tonnes of D-galacturonic acid. In turn, equimolar conversion of this D-galacturonic acid to L-galactonic acid and, subsequently, to L-ascorbic acid could be theoretically achieved. Therefore, even after factoring in conversion efficiencies below 100%, CPW could easily supply the raw material for world L-ascorbic acid production. The production of polymers derived from L-galactonic acid has also been studied (Romero Zaliz and Varela [2003], Romero Zaliz and Varela [2005]).

**Figure 1:**
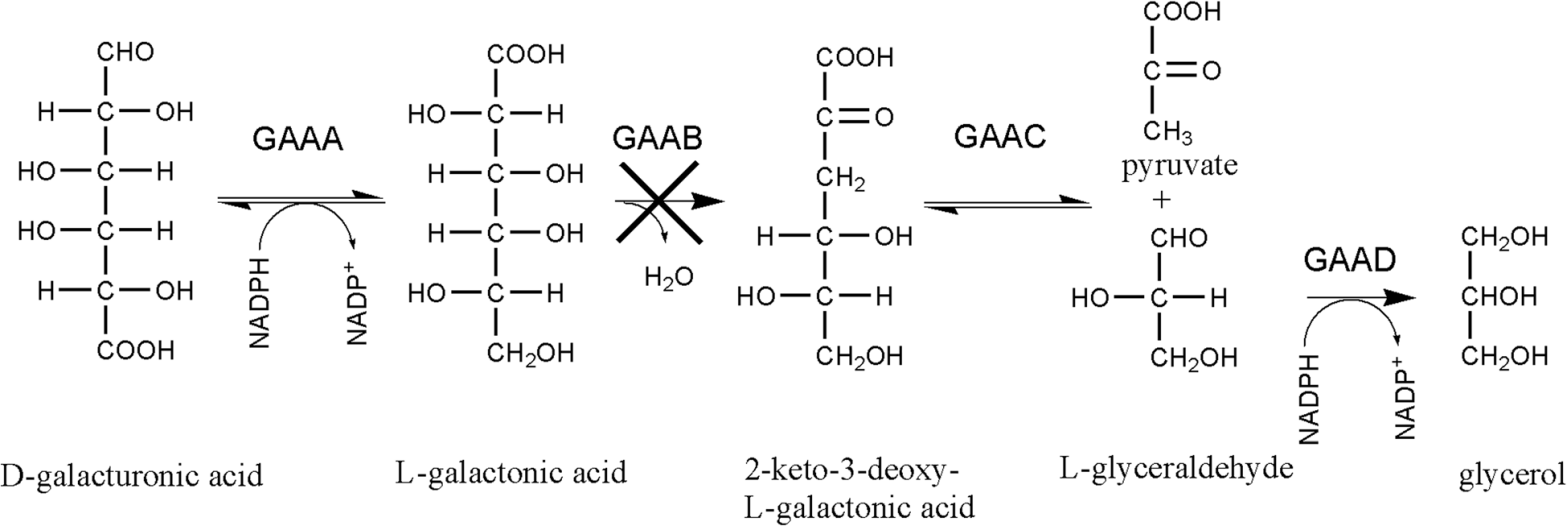
**The catabolic D-galacturonic acid pathway in*A. niger*.** The enzymes of this pathway are D-galacturonic acid reductase (GAAA), L-galactonate dehydratase (GAAB), 2-keto-3-deoxy-L-galactonate aldolase (GAAC) and L-glyceraldehyde reductase (GAAD). The strains used in the present work were engineered to have no GAAB activity.

L-Galactonic acid is an intermediate of the reductive pathway for catabolism of D-galacturonate that is found in moulds such as *Aspergillus niger* (Figure 1) (Richard and Hilditch [2009]). In *A. niger*, D-galacturonate is first reduced to L-galactonate by the D-galacturonate reductase, GAAA (Martens-Uzunova and Schaap [2008]). L-Galactonate is subsequently converted to 2-keto-3-deoxy-L-galactonate (3-deoxy-L-threo-hex-2-ulosonate) by the L-galactonate dehydratase, GAAB, which is then further metabolized to pyruvate and glycerol by the action of GAAC and GAAD (Martens-Uzunova and Schaap [2008]).

The deletion of the gene coding for the L-galactonate dehydratase, *gaaB*, resulted in a strain of *A. niger* that does not grow on D-galacturonic acid but instead converts D-galacturonate to L-galactonate (Kuivanen et al. [2012]). Since this strain produces pectinases, it was able to produce L-galactonate from polygalacturonate in a submerged fermentation (Kuivanen et al. [2012]). The aim of the current work was to determine whether this strain could be used to hydrolyse the pectin in the CPW and convert the resulting D-galacturonic acid to L-galactonic acid in a single fermentation process.

## Materials and methods

### Strains

The *Aspergillus niger* strain ATCC 1015 (CBS 113.46) was used as a wild type. Engineered strains ATCC 1015 Δ*gaaB* (L-galactonate dehydratase deletion strain) and Δ*gaaB*-*gaaA* (L-galactonate dehydratase strain with overexpression of the D-galacturonate reductase *gaaA*) were described previously (Kuivanen et al. [2012]). The strains Δ*gaaB* and Δ*gaaB*-*gaaA* are deposited in VTT Culture Collection as D-121454 and D-121455, respectively.

### Preparation of the substrate

Citrus processing waste (CPW), remaining after the preparation of fresh orange juice, was obtained from a local restaurant in Curitiba (Brazil). The CPW was dried at 60°C and milled with a grinder to a particle size of 0.9-2.4 mm. The ground CPW was washed with water in order to remove soluble sugars and dried again at 60°C. Finally, the powder was autoclaved for 40 min at 120°C.

### Submerged fermentations

For the submerged fermentations (SmF) with CPW, mycelia were pre-grown in 250-ml Erlenmeyer flasks in a medium containing (in g l^−1^): yeast extract 10, peptone 20 and gelatin 30. Pre-cultures were inoculated with a spore suspension and incubated overnight at 28°C, 200 rpm. Mycelia were harvested by vacuum filtration and rinsed with sterile water. Submerged cultures contained 40 g l^−1^ of the prepared CPW, which, due to the residual water, represents 35.1 g l^−1^ on a dry mass (DM) basis and were supplemented with *A. nidulans* defined minimal medium (Barratt et al. [1965]), containing (in g l^−1^): NaNO_3_ 6, KCl 0.52, MgCl_2_ 0.52 and KH_2_PO_4_ 1.52. In a separate culture, only sterile water with 40 g l^−1^ of the prepared CPW was used. Cultures were carried out in 250-ml Erlenmeyer flasks containing 50 ml final volume and were inoculated with 10 g l^−1^ (2.3 g l^−1^ DM) of pre-grown mycelia and incubated at 28°C and at 200 rpm. Samples were taken for HPLC analysis at intervals and solid matter (mycelia + insoluble substrate) was removed by centrifugation. In the fermentation for purification of the L-galactonic acid, 40 g l^−1^ of the prepared CPW was fermented in 500 ml of water in a 2000 ml flask. The culture was inoculated and incubated as described above.

### Solid-state fermentations

For the small scale solid-state fermentation (SSF) 2 g (1.76 g DM) of the CPW, prepared as described above, was added to each 100-ml Erlenmeyer flask. Sterile water (6 ml) containing inoculum of 2×10^7^ spores was added. The flasks were incubated at 28°C and a relative humidity of 96%. The effect of nutritional supplementation in SSF was studied by adding either 6 ml of pure water or a solution containing (in g l^−1^): K_2_HPO_4_ 3, (NH_4_)_2_SO_4_ 13, MgSO_4_.7H_2_O 5, KCl 10 and FeSO_4_.7H_2_O 0.1.

In order to investigate the potential for scaling up the process, packed-bed bioreactors (28 cm by 3.5 cm, ~270 ml) were used. For each column, 10 g (8.78 g DM) of the prepared CPW was wetted with 30 ml of the nutritional supplementation (as above) containing an inoculum (10^7^ spores per g prepared CPW). Columns were incubated in a water bath maintained at 28°C. Each column received a flow rate of air (saturated with water at 28°C) of 150 ml min^−1^.

For the extraction of the fermentation products, 15 ml of sterile water were added per 1 g (0.88 g DM) of fermented solids and the suspension was incubated at 28°C and 200 rpm for an hour. The liquid was collected by vacuum filtration and analysed by HPLC.

### D-galacturonic acid content of the CPW

In order to estimate the total D-galacturonic acid content in the substrate, 1 g (0.88 g DM) of the prepared CPW was hydrolysed with 127 PGNU (polygalacturonase unit, as defined by Novozymes) per ml of a commercial pectinase (Pectinex Ultra SP-L, Novozymes) in a final volume of 30 ml. The reaction mixture was incubated overnight at 30°C and at 100 rpm. The hydrolysed orange peel suspension was centrifuged and the supernatant was analysed by HPLC. The water content in the dried CPW was determined by heating the CPW in oven at 100°C overnight and weighing to determine the loss of mass.

### Transcriptional analysis

For the transcriptional analysis in the submerged fermentations, 1 ml samples were collected and the mycelia were harvested by vacuum filtration, frozen with liquid nitrogen and stored at −80°C. RNA was extracted with the RNeasy Plant Mini Kit (Qiagen) following the manufacturer’s instructions.

cDNA was prepared using the First Strand cDNA Synthesis Kit (Roche) following the manufacturer’s instructions. Quantitative PCR was carried out using the LightCycler SYBR green I master mix and the LightCycler 96 System. The transcription of putative D-galacturonate transporters An14g04280 and An03g01620 (Martens-Uzunova and Schaap [2008]) was quantified with the primers listed previously (Kuivanen et al. [2012]). The transcription levels were normalized to actin using the accompanying software (Advanced Relative Quantification Tool).

### L-galactonic acid purification

After the 500-ml SmF, the broth was separated by vacuum filtration and loaded onto an ion-exchange resin (Dowex 1X8, formate form). The bound L-galactonic acid was eluted with a linear formic acid gradient from 0 to 3 M. Fractions were collected and analysed by HPLC. The fractions with the highest L-galactonic acid content were combined and lyophilized. The resulting powder was dissolved in water and the proportions of linear L-galactonic acid and L-galactono-1,4-lactone were quantified using the colorimetric lactone assay (Hestrin [1949]). In the assay, 200 μl of the dissolved product was mixed with 400 μl of solution containing 4 M hydroxylamine and 4 M NaOH, followed by acidification with 200 μl of 4 M HCl and addition of 200 μl of 100 g l^−1^ FeCl_3_ in 0.1 M HCl. The absorbance was measured at 540 nm. Commercial L-galactono-1,4-lactone (Sigma) was used for the standard curve.

In an alternative procedure, the suitability of calcium precipitation for the purification was studied, using a method similar to that described for succinate (Li et al. [2010b]). In order to remove some of the impurities and clarify the broth, filtered final broth was stirred with activated carbon (12.5% w/v) for an hour. The activated carbon was then removed by filtration and the resulting clear liquid was collected. In order to precipitate the product as calcium L-galactonate, the liquid was stirred and solid Ca(OH)_2_ was added until the pH rose to 12.6. The resulting precipitate was collected by filtration and rinsed with cold water. L-Galactonic acid was released from the calcium salt by adding 0.5 M H_2_SO_4_ until pH 2.0. The precipitate (CaSO_4_) was removed by filtration and the filtrate was analysed by HPLC.

### HPLC analysis and calculations

The concentrations of sugars and sugar acids were determined by HPLC, using a fast acid analysis column (100 by 7.8 mm, Bio-Rad laboratories, Hercules, CA) linked to an Animex HPX-87H organic acid analysis column (300 by 7.8 mm, Bio-Rad Laboratories), with 5 mM H_2_SO_4_ as the eluent and a flow rate of 0.5 ml min^−1^. The column was maintained at 55°C. Peaks were detected with a Waters 410 differential refractometer.

**Figure 2:**
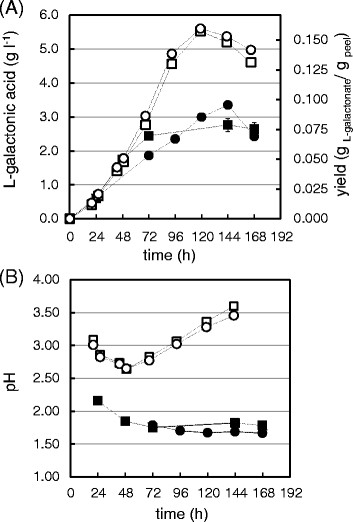
**The engineered strains*∆gaaB*(squares) and*∆gaaB*-*gaaA*(circles) grown on CPW in SmFs with (open symbols) and without (solid symbols) nutritional supplementation.(A)** L-Galactonic acid production, **(B)** the pH over the course of the fermentation. The production presented on a DM basis, error bars represents ± SEM, n = 3, if not visible then they are smaller than the symbol.

**Figure 3:**
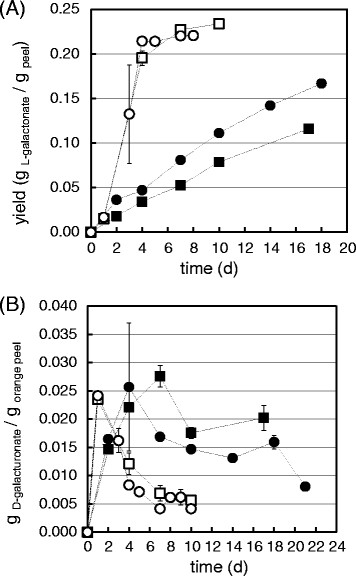
**The engineered strains*∆gaaB*(squares) and*∆gaaB*-*gaaA*(circles) grown on CPW in SSFs with (open symbols) and without (solid symbols) nutritional supplementation.(A)** L-Galactonic acid production, **(B)** the released D-galacturonic acid over the course of the fermentation. The production presented on a DM basis, error bars represents ± SEM, n = 3 without the salt supplementation and n = 2 with the salt supplementation, if not visible then they are smaller than the symbol.

All the sugar and sugar acid contents, the product yields and production rates were calculated on the basis of the DM of CPW used. Margins of error are presented as standard errors of the means (SEM). All the statistical tests were carried out using Student’s t-test. In the Table 1, the “product yield” is calculated from the highest L-galactonic acid concentration during the fermentation (Figures 2A and 3A). The “product yield of theoretical maximum” represents the ratio of the highest L-galactonic acid content (in the fermentation) to the D-galacturonic acid content in the CPW (determined as described above).

**Table 1:** Initial productivities, product yields and product yields as a percentage of the theoretical maximum from SmFs and SSFs on a DM basis

**Strain**	**Initial productivity**		**Product yield, Y**_**p/s**_		**Product yield (%) of theoretical maximum**	
	**mg**_**L-galactonate**_**/g**_**peel**_**/h**		**mg**_**L-galactonate/**_**g**_**peel**_		**g**_**L-galactonate/**_**g**_**D-galacturonate**_	
	**SmF**	**SSF**	**SmF**	**SSF**	**SmF**	**SSF**
	**0-70.5 ± 1.5 h**	**0-96 h**				
**Δ*gaaB*** (without suppl.)	1.01 ± 0.04^c^	0.35 ± 0.01	79 ± 5	116 ± 2^c^	23%	43%
**Δ*gaaB-gaaA*** (without suppl.)	0.74 ± 0.03^c^	0.49 ± 0.02	95 ± 3	167 ± 2^c^	35%	62%
**Δ*gaaB*** (with suppl.)	1.16 ± 0.01	^d^2.14 ± 0.09	157 ± 3	^d^233 ± 2^c^	58%	87%
**Δ*gaaB-gaaA*** (with suppl.)	1.26 ± 0.02	^d^2.35 ± 0.03^c^	159 ± 3	^d^221 ± 6^c^	59%	82%

^c^The process type (SmF or SFF) significantly (p < 0.05) better than the other in the same nutritional conditions.

^d^Errors present ± SEM, n = 2.

Errors represent ± SEM, n = 3.

## Results

In order to investigate the conversion of CPW to L-galactonic acid, we inoculated ground CPW with wild type and engineered *A. niger* strains (*∆gaaB* and *∆gaaB*-*gaaA*) and tested solid-state (SSF) and submerged (SmF) fermentations. A key value-added by-product from CPW is D-limonene, which is inhibitory for many microbes (Pourbafrani et al. [2010]), including *A. niger* (Sharma and Tripathi [2008]). In our process, it was not specifically extracted, although the washing of CPW possibly decreased its D-limonene content. The inhibitory effect of D-limonene was not studied in the process. After the washing, the insoluble material, containing mainly pectin, cellulose and hemicellulose, was fermented in SmF and SSF processes. The D-galacturonic acid resulting from the pectin hydrolysis was reduced to L-galactonic acid by the Δ*gaaB* and Δ*gaaB*-*gaaA* strains. L-Galactonic acid production was not observed in either process using the wild type strain.

### Composition of CPW

The content of D-galacturonic acid in the substrate was analysed by hydrolysing the prepared CPW with pectinases. The D-galacturonic acid was quantified by HPLC. This allowed calculation of the maximal theoretical yield of L-galactonic acid that can be obtained from CPW (1 mol of L-galactonic acid per 1 mol of D-galacturonic acid). The water content in the prepared dry CPW substrate was 12.2 ± 0.4%. In the remaining matter, the content of free D-galacturonic acid was 270 ± 7 mg per g CPW (on a DM basis) after pectinase hydrolysis. The other main soluble components in the hydrolysed CPW were, in mg per g CPW, D-glucose 55 ± 1, D-galactose 62 ± 1, L-arabinose 67 ± 2 and L-rhamnose 15 ± 1. The soluble sugars and sugar acids released in the pectinase hydrolysis accounted for 46.6% of the dry mass of the CPW.

### Consolidated conversion of orange peel to L-galactonic acid in submerged fermentation

In SmF, supplementation with the mineral salt solution improved both the initial productivity and the final yield of L-galactonic acid for both *∆gaaB* and *∆gaaB-gaaA* (Table 1 and Figure 2A). The initial productivity increased from values of 0.74-1.01 mg g^−1^ h^−1^ to around 1.2 mg g^−1^ h^−1^ and the product yield increased from values of 79–95 mg g^−1^ to around 160 mg g^−1^. The highest L-galactonic acid titer among SmFs, 5.60 ± 0.09 g l^−1^ (after 119 h), was obtained with *∆gaaB-gaaA* in the supplemented process (Figure 2A). The pH during the fermentation was lower in SmFs without supplementation (Figure 2B). Although low pH increased L-galactonic acid production in SmF in a previous study (Kuivanen et al. [2012]), the value below 2 that was reached in the current study might have had a negative effect.

**Figure 4:**
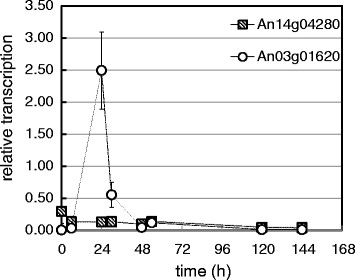
**Transcription of the putative D-galacturonate transporters An14g04280 and An03g01620 in*∆gaaB*strain over the course of the SmF on CPW.** The transcription levels are presented as relative to the transcription of actin. Error bars represents ± SEM, n = 3, at 24 h for An03g01620 n = 2, if not visible then they are smaller than the symbol.

In order to investigate the transport of the substrate into the cell, the transcription of two putative D-galacturonic acid transporters, An14g04280 and An03g01620, was followed by qPCR in the SmFs carried out with *∆gaaB* without the nutritional supplementation (Figure 4). The transcription of An14g04280 in *∆gaaB* did not show any induction on CPW, whereas the transcription of An03g01620 was clearly induced.

### Consolidated conversion of orange peel to L-galactonic acid in solid-state fermentation

As with SmF, in SSF supplementation with the mineral salts solution improved both the initial productivity and the final yield of L-galactonic acid for both *∆gaaB* and Δ*gaaB*-*gaaA* (Table 1 and Figure 3A). The initial productivity increased from values of 0.35-0.49 mg g^−1^ h^−1^ to values of 2.14-2.35 mg g^−1^ h^−1^ and the product yield increased from values of 116–167 mg g^−1^ to values 220–230 mg g^−1^. In the SSFs, the D-galacturonic acid profiles were also determined (Figure 3B). The rates of release and consumption of D-galacturonic acid were both faster in the supplemented SSF. In addition, both of the engineered strains produced lower D-galacturonic acid concentrations during the fermentation when compared to the wild type (wild type data not shown).

In large-scale SSF processes, packed-bed bioreactors are often used since they are less labour-intensive than tray-type processes (such as that undertaken in the current work in the Erlenmeyer flasks). In order to assess the performance of the process in this type of bioreactor, glass columns were loaded with 10 g (8.78 g DM) of CPW and the nutritional supplementation was used. The product yields were slightly lower than those in flask-scale SSFs: after 4 days, the L-galactonic acid yields obtained with *∆gaaB* and Δ*gaaB*-*gaaA* were 166 and 153 mg g^−1^, respectively, corresponding to 61 and 57%, respectively, of the theoretical maximum yields.

### Purification and applications of L-galactonic acid

L-Galactonic acid produced from CPW in a 500-ml SmF was purified using a Dowex resin and lyophilisation. Seventeen percent of the L-galactonic acid in the fermentation broth was recovered. In this purified product, 69% of the L-galactonic acid was in the linear form, whereas 31% was present as L-galactono-1,4-lactone. The alternative method for L-galactonic acid purification involved treatment of the fermentation broth with activated carbon then precipitation of L-galactonic acid as a calcium carboxylate salt. After treatment of the salt with sulphuric acid, the final liquid contained 20% of the initial L-galactonic acid.

## Discussion

There are several previous reports that describe processes converting pectin-rich biomass to ethanol using *Saccharomyces cerevisiae* or *Escherichia coli* (Edwards and Doran-Peterson [2012]). Due to the inability of these organisms to hydrolyse pectin and cellulose, additional pectinolytic and cellulolytic enzymes are required. From that perspective, it is an advantage to use a production organism, such as *A. niger*, that is naturally capable of hydrolysing pectin and cellulose: this enables a consolidated bioprocess. In the current work, we established a new consolidated bioprocess converting CPW to L-galactonic acid.

In the present study, the volumetric titers of L-galactonic acid after 72 h in the supplemented SmFs with Δ*gaaB* and Δ*gaaB*-*gaaA* were around 3 g l^−1^ resulting in an approximate volumetric productivity of 42 mg l^−1^ h^−1^ (Figure 2A). This value is relatively close to the initial productivities of 46–64 and 54–70 mg l^−1^ h^−1^ that were obtained with Δ*gaaB* and Δ*gaaB*-*gaaA*, respectively, in SmF of a defined medium supplemented with 10 g l^−1^ of D-galacturonic acid and 2 g l^−1^ of D-xylose (Kuivanen et al. [2012]). The product yields (% of theoretical maximum, i.e. g L-galactonic acid per g initial D-galacturonic acid) obtained in the defined conditions were 47 and 59% with Δ*gaaB* and Δ*gaaB*-*gaaA*, respectively (Kuivanen et al. [2012]). The corresponding values in this study were similar, being 58 and 59% respectively (Table 1). However, the L-galactonic acid yields, 87 and 82% of the theoretical maximum, obtained in SSF with Δ*gaaB* and Δ*gaaB*-*gaaA*, respectively, in the current study, were clearly higher when compared to those of the SmFs (Table 1). The SSFs in packed-bed column bioreactors resulted in yields close to those of achieved in SmF. However, the longest bioreactor fermentation was extracted after 4 days, which may be too short a time for the optimal yield.

The higher product yields in the SSFs might be due to a difference in pectin hydrolysis under submerged and solid-state conditions. In fact, the presence of free sugars, such as D-glucose or sucrose, strongly decreases the production of endo- and exo-pectinases by *A. niger* in SmF, but not in SSF, which indicates a possible difference in the regulation of pectin metabolism between submerged and solid-state conditions (Solis-Pereira et al. [1993]). This is most likely due to the concentration gradients that arise, with the concentrations of soluble sugars remaining low in the vicinity of the hyphae and therefore not causing catabolite repression (Viniegra-González and Favela-Torres [2006]).

Small amounts of D-galacturonic acid were still present in the SSFs after the L-galactonic acid production had slowed significantly (compare profiles in Figure 3A and B over the period of 72–216 h). In contrast, no D-galacturonic acid was found at the end of the SmFs (i.e. after 120 h, data not shown), even though the yields of L-galactonic acid were only 23–59% of the theoretical maximum values (Table 1). There are two possible explanations for the final product yields being below 100%: pectin hydrolysis may have been incomplete or some of the L-galactonic acid that was produced may have been consumed. The strain *∆gaaB* was unable to catabolize L-galactonic acid in submerged cultures when pure D-galacturonic acid or polygalacturonate were used (Kuivanen et al. [2012]). However, several additional carbon sources are present in the CPW and might induce expression of unspecific dehydratases, allowing some L-galactonic acid consumption, since the genome of *A. niger* contains at least four putative dehydratase coding genes in addition to *gaaB*. A similar kind of phenomenon was described in an engineered *A. niger* strain with a disturbed D-galacturonate pathway (a deletion of D-galacturonate reductase, *gaaA*) and an introduced uronic acid dehydrogenase: The resulting strain was able to utilize D-galacturonate through an unknown pathway (Mojzita et al. [2010]).

The transcription of two putative D-galacturonate transporters An14g04280 and An03g01620 was followed during the SmF without the nutritional supplementation (Figure 4). These transporters have a strong similarity to hexose transporters and their transcription is induced in wild type *A. niger* when cultured on D-galacturonate, polygalacturonate and sugar beet pectin (Kuivanen et al. [2012], Martens-Uzunova and Schaap [2008]). In the present study, only induction of An03g01620 was observed. This transcriptional pattern is similar to that obtained when *A. niger ∆gaaB* was cultivated on a mixture of pure D-galacturonic acid and D-xylose (Kuivanen et al. [2012]). The reason for altered expression of An14g04280 in *∆gaaB* remains unclear, however, it may have a negative effect on the D-galacturonic acid transport into the cell. The peak of the An03g01620 induction during growth on CPW occurred in the current work at 24 h whereas the corresponding peak on pure D-galacturonic acid was at 3 h (Kuivanen et al. [2012]). The time difference is most likely due to the inhibitory effect of other sugars of the CPW on pectin hydrolysis and D-galacturonic acid catabolism at the beginning of the SmF.

In the context of L-ascorbic acid (vitamin C) synthesis, it is advantageous to know whether the product from the consolidated process is linear L-galactonic acid or L-galactono-1,4-lactone: a single reduction step is required to form L-ascorbic acid from the lactone, whereas the linear form would need to be first lactonized. The L-galactonic acid recovered from the Dowex resin was a mixture of linear and lactonized form. The lactonization of L-galactonic acid is thermodynamically more favourable at low pH, thus the elution with formic acid probably caused the partial lactonization. We also tested the suitability of calcium precipitation for the L-galactonic acid recovery because it is widely used in the industry. The purification yield remained relatively low (20%) since the process was not optimized for L-galactonic acid. However, this method, once optimized, could potentially be used for L-galactonic acid recovery.

Currently, industrial production of L-ascorbic acid begins with D-sorbitol, which is produced by the hydrogenation of D-glucose derived from corn or wheat (Bremus et al. [2006]). The predominant manufacturing methods are still based on Reichstein process, which is a chemical process (Reichstein and Grüssner [1934]). Current processes, that also involve fermentation steps, are still energy intensive (Bremus et al. [2006]) and use valuable D-glucose as a raw material. From that perspective, a production method utilizing a single fermentation step and low value CPW as the raw material would be highly desirable.

In the present work, L-galactonic acid was produced from CPW in a consolidated process by engineered *A. niger* strains. Close to 90% product yield was achieved, based on the D-galacturonic acid contained in the pectin of the CPW. To the best of our knowledge, this is the first report on a consolidated bioprocess in which a pectin-rich residue is converted to a high value biochemical using an engineered microorganism.

## Competing interests

The authors declare that they have no competing interests.
